# Supplementary material for: Leveraging Immunofocusing and Virus-like Particle Display to Enhance Antibody Responses to the Malaria Blood-Stage Invasion Complex Antigen PfCyRPA
Source: Vaccines (Basel). 2024 Jul 30;12(8):859. doi: 10.3390/vaccines12080859 (PMC11359962; doi:10.3390/vaccines12080859)
Supplement: Supplementary file 1 [file vaccines-12-00859-s001.zip › vaccines-3092161-supplementary.pdf]

Supplementary Figures

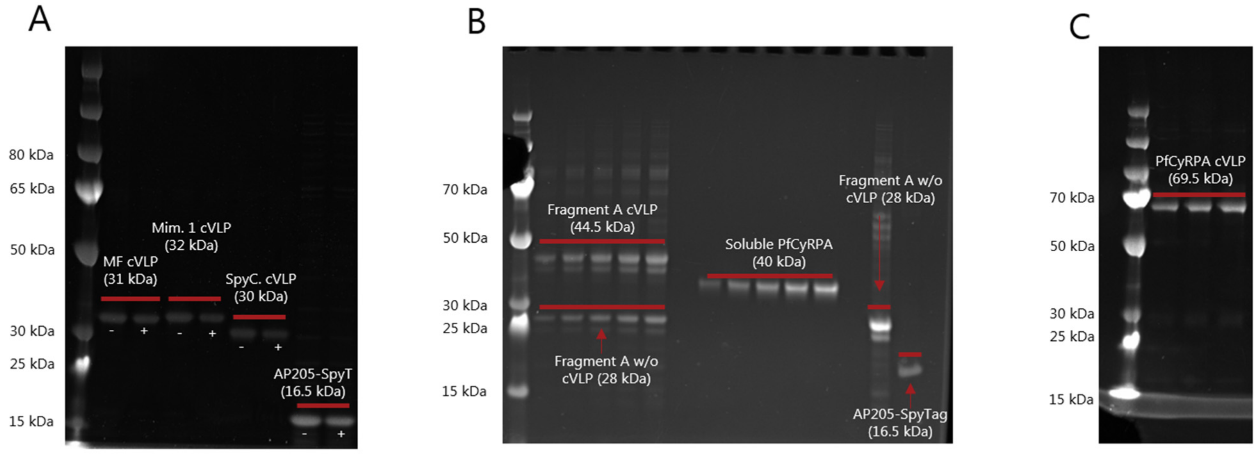

Figure S1. Coupling efficacy of cVLP vaccines. (A) MF, Mimotope 1 and SpyCatcher cVLPs as well as the AP205-SpyT subunit. '-' and '+' signifies without or with heavy centrifugation to examine for precipitation. (B) Fragment A cVLP. Repetition of lanes is due to varying amounts of protein having been loaded. Shows Fragment A cVLP, soluble PfCyRPA, uncoupled Fragment A, and uncoupled AP205-SpyTag. (C) PfCyRPA cVLP. Varying protein amounts loaded.

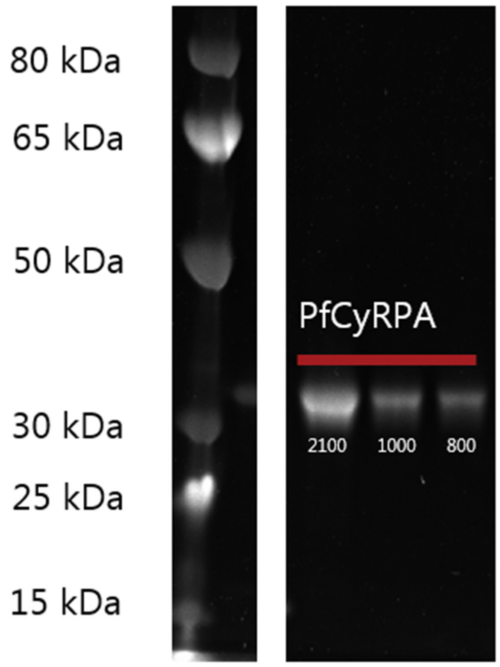

Figure S2. Purity of PfCyRPA. 2100, 1000 and 800 µg of protein was analyzed using SDS-PAGE. The protein has an expected size of 40 kDa.

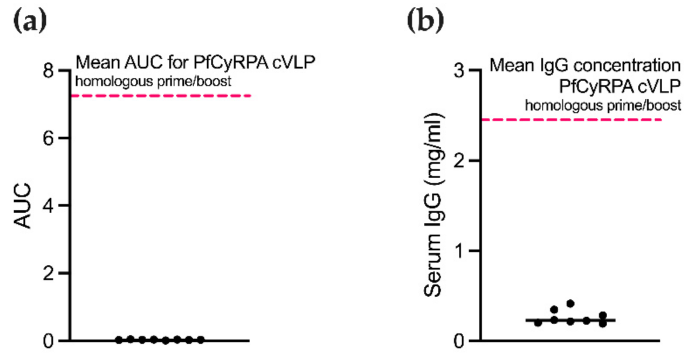

Figure S3. PfCyRPA response and serum IgG concentration of prebleeds prior to immunizations. (a): AUC of serum titrations of prebleeds prior to vaccinations from eight mice. Mean AUC of homologous prime/boost with PfCyRPA cVLP as seen in figure 2E depicted as pink line. (b): Serum IgG concentrations of prebleeds prior to vaccinations from eight mice. Mean IgG concentration of homologous prime/boost with PfCyRPA cVLP as seen in figure 2F depicted as pink line.

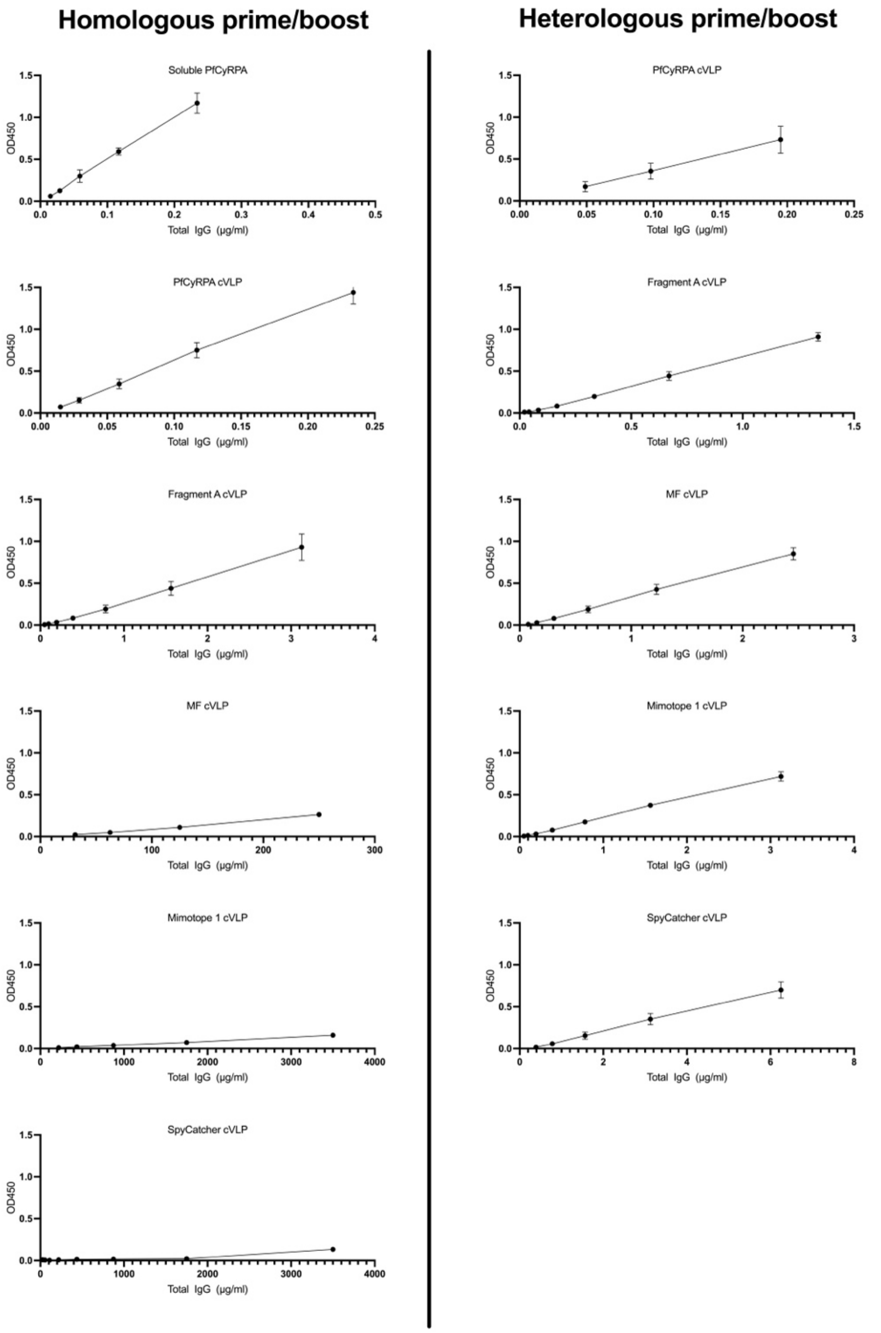

Figure S4. Determining relative amount of PfCyRPA specific antibody in IgG pool through ELISA. Total IgG in µg/ml on x-axis, OD450 readout with background subtracted on y-axis. Linear regression depicted as line.

Table S1. Overview of vaccination groups. Vaccine used for prime and boost, as well as the mass and number of moles per injection, is reported. Each group consisted of eight mice. Five µg of soluble PfCyRPA and 5 µg of PfCyRPA cVLP was used. For the other cVLPs, equal moles of cVLPs as the PfCyRPA cVLP was used, resulting in different protein masses for vaccinations. All mice were female BALB/c mice.

	Group #	# of mice	Prime	Mass (µg)	Moles (pmol)	Boost	Mass (µg)	Moles (pmol)
Homologous prime/boost	1	8	Soluble PfCyRPA	5	125	Soluble PfCyRPA	5	125
	2	8	PfCyRPA cVLP	5	0.4	PfCyRPA cVLP	5	0.4
	3	8	Fragment A cVLP	3.2	0.4	Fragment A cVLP	3.2	0.4
	4	8	MF cVLP	2.3	0.4	MF cVLP	2.3	0.4
	5	8	Mimotope cVLP	2.3	0.4	Mimotope cVLP	2.3	0.4
	6	8	SpyCatcher cVLP	2.1	0.4	SpyCatcher cVLP	2.1	0.4
Heterologous prime/boost	7	8	Soluble PfCyRPA	5	125	PfCyRPA cVLP	5	0.4
	8	8	Soluble PfCyRPA	5	125	Fragment A cVLP	3.2	0.4
	9	8	Soluble PfCyRPA	5	125	MF cVLP	2.3	0.4
	10	8	Soluble PfCyRPA	5	125	Mimotope cVLP	2.3	0.4
	11	8	Soluble PfCyRPA	5	125	SpyCatcher cVLP	2.1	0.4
